# Treatment of idiopathic hypersomnia with modafinil in an individual at clinical high risk for psychosis: A case report

**DOI:** 10.1002/pcn5.70115

**Published:** 2025-05-05

**Authors:** Yutaro Sato, Atsushi Sakuma, Hiroaki Tomita

**Affiliations:** ^1^ Department of Psychiatry Tohoku University Hospital Sendai Japan; ^2^ National Hospital Organization Sendai Medical Center Sendai Japan; ^3^ Department of Psychiatry Tohoku University Graduate School of Medicine Sendai Japan

**Keywords:** clinical high risk for psychosis, idiopathic hypersomnia, modafinil, multiple sleep latency test, polysomnography

## Abstract

**Background:**

Recent studies have shown that sleep disturbances are frequent at different stages of psychosis, including clinical high risk for psychosis (CHR‐P). However, the comorbidity of hypersomnia with CHR‐P and its treatment have rarely been reported or discussed.

**Case Presentation:**

A 16‐year‐old female diagnosed with major depressive disorder and CHR‐P experienced worsening excessive daytime sleepiness (EDS), including falling sleep in class. Polysomnography and multiple sleep latency tests confirmed the diagnosis of idiopathic hypersomnia (IH), and treatment with modafinil (100 mg/day) was initiated. EDS improved after increasing the modafinil dose to 200 mg/day. No side effects or exacerbations of psychotic symptoms were observed. EDS recurred after she entered university and was treated with 300 mg/day of modafinil without side effects or transition to psychosis.

**Conclusion:**

This case demonstrates modafinil's efficacy and safety in treating IH in individuals with CHR‐P. However, whether modafinil use increases the risk of transition to psychosis remains unclear. Further research is required on modafinil as a treatment for hypersomnia in individuals with CHR‐P.

## BACKGROUND

Sleep disturbances are observed throughout the course of psychosis, including in individuals at clinical high risk for psychosis (CHR‐P).^1^, ^2^ Sleep quality is poorer in those with CHR‐P than in healthy controls.^2^ Sleep disturbances have been associated with worsening CHR‐P symptoms.^1^ However, few studies have investigated excessive daytime sleepiness (EDS) or hypersomnia during the early stages of psychosis. Notably, 23.3% of patients with early psychosis screen positive for excessive sleepiness disorder.^3^ EDS is associated with low activity levels during early psychosis.^4^ The comorbidity of hypersomnia with CHR‐P and treatment for hypersomnia in individuals with CHR‐P have rarely been reported or discussed. Here, we report a case of idiopathic hypersomnia (IH) in a patient with CHR‐P.

## CASE PRESENTATION

The patient was a 16‐year‐old female high school student. Neurodevelopmental disorders were not indicated. Since elementary school, she had persecutory ideas and hallucinations. She experienced feelings of being watched by people on the train or in the store and thoughts of being stabbed or kidnapped while walking down the street. She experienced auditory hallucinations, such as hearing a car door close near home, and visual hallucinations, such as seeing human shadows before falling asleep or in dim places. At the age of 16 years, she exhibited persistent depressive symptoms, including depressed mood, loss of interest, insomnia, diminished ability to concentrate, fatigue, feelings of guilt, and suicidal ideation, and was diagnosed with major depressive disorder. Her persecutory ideas and hallucinations worsened with the onset of depression, and fulfilled the criteria for CHR‐P according to the Comprehensive Assessment of At‐Risk Mental State,^5^, ^6^ confirming the diagnosis. She was initially treated with sertraline at 25 mg/day. No effect of sertraline on sleepiness was observed. At the beginning of the next semester, she complained of falling asleep during class and reported that her sleepiness had been present since entering high school but had recently worsened. She had extreme sleepiness during the day, especially in the morning. Sleep diaries indicated that EDS occurred almost daily, including on holidays. Naps did not alleviate EDS. Cataplexy and sleep paralysis were not observed. Her persecutory ideas and hallucinations had improved without the use of antipsychotics. She had difficulty falling asleep at night and was prescribed lemborexant up to 10 mg/day. Sertraline was increased to 50 mg/day, and her depression improved. Lemborexant and sertraline did not induce daytime sleepiness. She scored 16 points on the Pittsburgh Sleep Quality Index (PSQI) and 17 points on the Epworth Sleepiness Scale (ESS). Nocturnal polysomnography (PSG) and multiple sleep latency tests (MSLT) were performed. PSG revealed no sleep‐onset rapid eye movement period (SOREMP), and sleep architecture was preserved (Table 1). The apnea–hypopnea index was 0.2, which was not indicative of obstructive sleep apnea. MSLT showed a mean sleep latency of 3.75 min and no SOREMP. She was diagnosed with IH based on the International Classification of Sleep Disorders, Third Edition.^7^ Modafinil was started at 100 mg/day after explaining its potential risks to the patient, including worsening psychotic symptoms, and a shared decision‐making process that considered the risks and benefits was conducted. No specific side effects or exacerbation of psychotic symptoms were observed, and EDS was reduced. The PSQI and ESS scores decreased to 12 and 15 points, respectively. When the modafinil dose was increased to 200 mg/day, the patient stopped falling asleep in class. The remaining depressive symptoms, including depressed mood, improved after sertraline dose was increased to 50 mg/day.

**Table 1:** Polysomnography results.

Index	Value
Total recording time (min)	841.0
Total time in bed (min)	510.6
Total sleep time (min)	489.3
Sleep efficiency (%)	95.8
Sleep latency (min)	5.3
REM latency (min)	103.0
Wake after sleep onset (min)	16.0
Stage N1 sleep (% of TST)	1.5
Stage N2 sleep (% of TST)	58.1
Stage N3 sleep (% of TST)	23.7
REM sleep (% of TST)	16.7
Apnea/hypopnea index (/h)	0.2
Mean SpO_2_ (%)	98
Minimum SpO_2_ (%)	94
Periodic leg movement index (/h)	0.9

Abbreviations: REM, rapid eye movement; SpO_2_, percutaneous oxygen saturation; TST, total sleep time.

**Figure 1:**
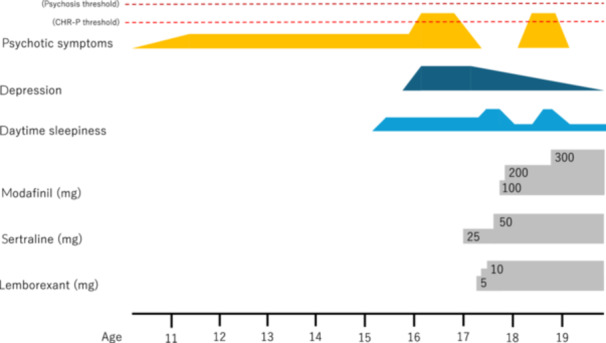
Clinical course of the case, showing the timeline of symptoms and treatment. Psychotic symptoms do not exceed the threshold for psychosis. CHR‐P, clinical high risk for psychosis.

After entering university, she had recurring episodes of being watched by people and persecutory ideas of being stabbed; however, she remained at the CHR‐P and did not transition to psychosis. She had no complaints of insomnia, and no recurrence of depression was observed. About 6 months after entering university, EDS occurred during class, and the modafinil dose was increased to 300 mg/day without side effects or worsening psychotic symptoms. EDS reduced, and she continued to attend university and work part‐time. The clinical course of the case, including the details of pharmacotherapy, is shown in Figure 1.

## DISCUSSION

Our patient was initially diagnosed with depression and CHR‐P. PSG and MSLT were performed because of complaints of sleepiness. She was diagnosed with IH comorbid with depression and CHR‐P, and modafinil improved EDS without worsening psychotic symptoms. To the best of our knowledge, this is the first case report in which IH was treated with modafinil in a patient with CHR‐P.

In our case, the patient had EDS since she entered high school; however, the diagnosis of IH was delayed. Delays in the diagnosis of IH have been reported, with difficulty in diagnosis being a major factor.^8^ Additionally, patients may not perceive EDS as a medical problem and often delay seeking medical services until it substantially affects their daily lives.^8^ Our case suggests that clinicians should pay attention to EDS for a proper diagnosis of hypersomnia.

In the diagnosis of IH, the assessment of drug‐induced sleepiness and the effect of drugs on PSG/MSLT results is important. The patient was taking sertraline and lemborexant at the time of PSG and MSLT, but adequate attention was paid to drug‐induced sleepiness, and no worsening of sleepiness due to these drugs was observed.

Modafinil is a wakefulness‐promoting agent used to treat narcolepsy and other sleep disorders. Its effects on negative symptoms and cognitive deficits in schizophrenia have been studied,^9^, ^10^ and a meta‐analysis reported that modafinil is neither better nor worse than placebo in preventing the worsening of psychosis, noting the low quality of the current evidence.^11^ No studies have specifically investigated the efficacy and safety of modafinil in individuals with CHR‐P, and whether modafinil increases the risk of transition to psychosis remains unclear.

Case reports have described psychosis associated with modafinil in patients with hypersomnia, including narcolepsy,^12^, ^13^, ^14^ IH,^15^ and Klein–Levin syndrome.^16^ Sahan et al. reported a case of IH with a background similar to ours: a 17‐year‐old female preparing for university entrance exams.^15^ In this case, modafinil was initiated at 100 mg/day and then increased to 200 mg/day. The patient attempted suicide due to stress and exam anxiety by overdosing on 12 g of modafinil, resulting in encephalopathy and persistent psychosis. Reports indicate that the use of psychostimulants as “cognitive enhancers” among students has increased in the United Kingdom, with lifetime use of modafinil exceeding that of methylphenidate or dexamphetamine.^17^ Clinicians should be cautious regarding the inappropriate use of modafinil, especially when the patient is a student seeking cognitive enhancement.

In our case, the recurrence of feelings of being watched did not occur immediately after the initiation or dose increase of modafinil. However, caution was warranted regarding the potential worsening of psychotic symptoms or the transition to psychosis. The risks and benefits of modafinil were explained to the patient, and shared decision‐making was performed. The reasons why modafinil did not induce psychosis in this case are worthy discussing. A meta‐analysis showed that baseline antidepressants exposure in CHR‐P was associated with a reduced risk of transition to psychosis.^18^ Antidepressants treatment for depression may have contributed to preventing the transition to psychosis in our case.

Management strategies for hypersomnia with psychotic symptoms are yet to be established. Hanin et al. conducted a systematic review of the relationship between narcolepsy and psychosis, and proposed a clinical algorithm for evaluating and treating narcolepsy with psychotic symptoms.^19^ In this algorithm, patients with narcolepsy and psychotic symptoms are classified into three groups: (i) those with typical hallucinations of narcolepsy, (ii) those with atypical narcolepsy and psychotic‐like symptoms, and (iii) those with narcolepsy and a comorbid psychotic disorder. Patients in any group may experience drug‐induced psychosis, especially those receiving high doses of psychostimulants or with a history of psychotic symptoms. Hanin et al.'s approach could be applied to the relationship between IH and psychosis; however, it requires accumulating cases of IH with psychosis.

Treatment options other than modafinil for IH need to be discussed. The American Academy of Sleep Medicine clinical practice guideline recommends modafinil and suggests clarithromycin, methylphenidate, pitolisant, and sodium oxybate for IH in adults.^20^ Only modafinil is given a “strong” recommendation, meaning that almost all patients should receive this medication. The guideline strongly favors modafinil due to the balance between desirable and undesirable effects. Although there are no established drug recommendations for IH in youth at CHR‐P, given the relative benefits and risks, modafinil is recommended.

This study had some limitations. First, this was a single case report. Our findings alone are not sufficient to establish the efficacy and safety of modafinil for IH in individuals at CHR‐P, and additional cases are needed in future studies. Second, further long‐term follow‐up may be required to confirm the transition to psychosis. We plan to follow the long‐term course of this case.

## CONCLUSION

We report a case of IH accompanied by CHR‐P in which IH was alleviated by modafinil without transition to psychosis, while modafinil has been suggested to have a risk of inducing psychosis. When modafinil is used for IH in individuals with CHR‐P, the risk of inducing psychosis and the benefit of improving IH should be carefully considered on a case‐by‐case basis. More cases need to be documented to establish treatment strategies, including modafinil prescription, for IH accompanied by CHR‐P.

## AUTHOR CONTRIBUTIONS

Yutaro Sato treated the patient and wrote and revised the manuscript. All authors participated in the discussions, wrote the manuscript, and approved the final manuscript.

## CONFLICT OF INTEREST STATEMENT

The authors declare no conflicts of interest.

## ETHICS APPROVAL STATEMENT

This case report was conducted in accordance with ethical guidelines for case reports of the Japanese Society of Psychiatry and Neurology.

## PATIENT CONSENT STATEMENT

Written informed consent was obtained from the patient for the publication of this report.

## CLINICAL TRIAL REGISTRATION

N/A.

## Data Availability

The data that support the findings of this study are available from the corresponding author upon reasonable request.
